# Tunnelled intrathecal catheter enabling repeated spinal anaesthesia in MR-Linac radiotherapy: a case report

**DOI:** 10.1186/s12871-026-04108-z

**Published:** 2026-08-06

**Authors:** Hasan Huseyin Kilic, Onur Bukagikiran

**Affiliations:** 1 Department of Anaesthesiology, Dogus University, Istanbul, Turkey; 2 Department of Anaesthesiology and Reanimation, Hisar Intercontinental Hospital, Istanbul, Turkey

**Keywords:** Anaesthesia management, Case report, Intrathecal catheter, MR-Linac, Radiotherapy, Spinal anaesthesia

## Abstract

**Background:**

Magnetic resonance-guided linear accelerators (MR-Linac) integrate magnetic resonance imaging (MRI) with radiotherapy delivery, enabling adaptive treatment. However, patient immobility is required for 60–90 min within a high-field magnet, making anaesthetic management challenging. Sedation may be inadequate in patients with opioid tolerance or obstructive sleep apnoea, while general anaesthesia (GA) poses significant risks due to airway and equipment limitations in the MR environment.

**Case presentation:**

We present a 78-year-old male with prostate cancer, severe chronic pain, opioid tolerance, and prior spinal instrumentation undergoing 28 MR-Linac sessions. Sedation attempts failed due to persistent involuntary leg movements and apnoea episodes. GA was deemed high-risk due to obesity, obstructive sleep apnoea, chronic opioid and alcohol use, and limited emergency access in the MR-Linac suite. Epidural anaesthesia was unfeasible due to adhesions from prior spinal surgery. A tunnelled intrathecal catheter was placed at the L3–L4 level, enabling daily intermittent spinal anaesthesia with 0.5% bupivacaine. All 28 fractions were completed uneventfully, without infection, neurological deficit, or catheter-related complications.

**Conclusion:**

Repeated spinal anaesthesia delivered through a tunnelled intrathecal catheter enabled successful completion of MR-Linac radiotherapy in this carefully selected patient after conventional sedation had failed. This case highlights an individualized management approach for a challenging clinical scenario.

**Supplementary Information:**

The online version contains supplementary material available at https://doi.org/10.1186/s12871-026-04108-z.

## Introduction

Magnetic resonance-guided linear accelerators (MR-Linac) integrate high-field magnetic resonance imaging (MRI) with radiotherapy delivery, enabling real-time imaging and adaptive treatment, particularly for soft tissue tumours such as prostate cancer [1–5]. The widespread adoption of MR-Linac systems has increased the demand for sedation or anaesthesia, as these procedures require patients to remain immobile in a supine position for 60–90 min within a 1.5–3 T magnetic field [3, 4].

Electromagnetic constraints restrict personnel access during irradiation, complicating immediate intervention in the event of cardiorespiratory compromise [1]. Consequently, airway management, haemodynamic monitoring, and emergency response become substantially more challenging [1, 5].

While general anaesthesia (GA) offers immobility, repeated administration over several weeks may lead to cumulative physiological stress, particularly in elderly or high-risk patients. Moreover, MR-compatible anaesthesia equipment is limited, posing additional logistical and safety concerns [5].

Although sedation is typically adequate for MRI, it may be insufficient in patients with severe involuntary movements, difficult airways, or obstructive sleep apnoea (OSA). In these scenarios, neuraxial techniques—predictable and haemodynamically stable—may offer a safer alternative.

Herein, we report a case in which standard sedation protocols failed to suppress involuntary movement in a patient undergoing MR-Linac therapy. Due to multiple anaesthetic limitations, a tunnelled intrathecal catheter was placed, enabling effective daily intermittent spinal anaesthesia and the successful completion of radiotherapy. Here, we describe the successful use of repeated spinal anaesthesia delivered through a tunnelled intrathecal catheter to facilitate MR-Linac radiotherapy in a patient for whom conventional sedation was unsuccessful.

## Case presentation

A 78-year-old male (BMI: 32) with localised prostate cancer was scheduled to undergo 28 MR-Linac sessions (one per weekday). His comorbidities included hypertension, OSA, chronic opioid use (due to pelvic bone metastasis–related pain), chronic alcohol consumption, and prior lumbar spinal instrumentation following vertebral fractures.

Across several attempts, various combinations of midazolam, propofol, fentanyl, and ketamine were trialled to suppress persistent involuntary leg movements in the supine position. The patient’s severe pelvic pain and opioid tolerance rendered these regimens ineffective. In addition, the severity of pelvic pain significantly limited mobility, and the patient frequently required wheelchair assistance. Escalation of sedation depth led to episodes of apnoea, making deeper sedation unsafe. Each failed session required rescheduling, significantly disrupting treatment continuity.

GA was deemed high-risk due to anticipated difficult airway, obesity, OSA, hypertension, chronic alcohol and opioid use, and the limited access inherent to the MR-Linac environment. Additionally, daily intubation and prolonged anaesthesia exposure were considered likely to impose cumulative physiological stress and increase complication risk.

Epidural anaesthesia via an indwelling catheter was initially considered but was not feasible due to spinal adhesions from prior instrumentation. A single-shot spinal anaesthesia using 2.5 mL of 0.5% bupivacaine was subsequently administered, resulting in effective anaesthesia and a successful MR-Linac session. This confirmed the feasibility of subarachnoid access despite altered spinal anatomy.

A tunnelled subarachnoid catheter (Perifix One, BRAUN^®^, Germany) was then placed under sterile conditions in the operating room at the L3–L4 level by a consultant anaesthesiologist with more than 10 years of experience in neuraxial anaesthesia. The procedure was performed using a conventional landmark-based technique without fluoroscopic or ultrasound guidance. Free flow of cerebrospinal fluid confirmed correct intrathecal placement before catheter advancement. Approximately 4 cm of the catheter was advanced into the subarachnoid space and tunnelled subcutaneously for approximately 12 cm before exiting the skin. The catheter was secured using a chlorhexidine gluconate-containing transparent dressing (3 M Tegaderm™ CHG), allowing daily inspection of the insertion site. For the first session, 2.5 mL of 0.5% bupivacaine was administered intrathecally, achieving a prolonged sensory block. In subsequent sessions, 2.0 mL proved sufficient.

During the initial five sessions, intravenous sedation with 2 mg midazolam was used. From the sixth session onward, no additional sedation was required as the patient adapted to the procedure.

All 28 sessions were completed successfully over 45 calendar days. No complications—such as infection, neurological deficit, catheter migration, meningeal irritation, or granuloma formation—were observed. The catheter was removed uneventfully following treatment completion.

## Discussion

Anaesthetic management in MR-Linac environments is uniquely challenging. Restricted access during beam-on phases complicates airway interventions and emergency responses [1]. In patients with OSA or altered airway anatomy requiring prolonged daily immobility, these constraints magnify the risks of apnoea, hypoventilation, and haemodynamic instability [5].

In our case, sedation protocols failed due to the patient’s chronic pain, opioid tolerance, and positional intolerance. GA was contraindicated due to multiple factors, including anticipated airway difficulty, OSA, and the burdens of daily intubation. Thus, alternative neuraxial strategies were explored.

Epidural catheterisation was unsuccessful due to adhesions from prior spinal surgery. This anatomical limitation led to the adoption of a subarachnoid approach.

The use of intrathecal catheters is well documented in chronic pain and oncology settings. Infection rates remain low (1–2%) when using tunnelled systems and strict aseptic technique [6, 7]. Nitescu et al. demonstrated the safety of long-term catheterisation in ambulatory pain management [8]. Dickson’s review further supports the risk–benefit profile of extended intrathecal therapy in select patients [9].

Granuloma formation, a rare but serious complication, is primarily associated with continuous intrathecal opioid administration [10]. Our approach involved only intermittent bolus doses of bupivacaine, thus likely minimising this risk. Li et al. also confirmed the safety of both single and dual intrathecal catheters in managing cancer pain, without significant neurological sequelae [11].

This technique allowed the safe, effective delivery of daily spinal anaesthesia without the need for systemic sedation or GA. The patient’s chronic pain was also alleviated during therapy, improving tolerability and compliance. While spinal anaesthesia and intrathecal catheterisation are well-established techniques, their application to repeated daily spinal anaesthesia over multiple weeks in the MR-Linac radiotherapy setting has not been previously reported. The novelty of this case therefore lies not in the anaesthetic technique itself, but in its successful adaptation to a clinically demanding scenario in which conventional sedation failed and repeated general anaesthesia was considered high risk.

Future prospective studies are warranted to better define patient selection criteria, optimal catheter management protocols, and the long-term safety of repeated spinal anaesthesia via tunnelled intrathecal catheters in prolonged radiotherapy settings.

## Conclusion

Successful completion of all planned MR-Linac sessions was achieved in this patient using repeated spinal anaesthesia delivered through a tunnelled intrathecal catheter after conventional sedation had failed. No catheter-related complications, neurological deficits, or infections were observed during treatment. This case highlights how individualized anaesthetic management enabled uninterrupted radiotherapy in a patient with complex clinical challenges.

## Supplementary Information

Supplementary Material 1.

## Data Availability

Not applicable.

## Abbreviations

BMI: Body mass index
GA: General anaesthesia
MRI: Magnetic resonance imaging
MR-Linac: Magnetic resonance–guided linear accelerator
OSA: Obstructive sleep apnoea
