# Supplementary material for: Tunnelled intrathecal catheter enabling repeated spinal anaesthesia in MR-Linac radiotherapy: a case report
Source: BMC Anesthesiol. 2026 Aug 6;26:654. doi: 10.1186/s12871-026-04108-z (PMC13729659; doi:10.1186/s12871-026-04108-z)
Supplement: Supplementary file 1 [file 12871_2026_4108_MOESM1_ESM.pdf]

CARE Checklist of information to include when writing a case report

CARE case report guidelines

(cc) BY-NC-ND

Topic	Item	Checklist item description	Reported on Line
**Title**	1	The diagnosis or intervention of primary focus followed by the words "case report"	Lines 1-2
**Key Words**	2	2 to 5 key words that identify diagnoses or interventions in this case report, including "case report"	Lines 31-33
**Abstract (no references)**	3a	Introduction: What is unique about this case and what does it add to the scientific literature?	Lines 10–15
	3b	Main symptoms and/or important clinical findings	Lines 17–25
	3c	The main diagnoses, therapeutic interventions, and outcomes	Lines 17–25
	3d	Conclusion—What is the main "take-away" lesson(s) from this case?	Lines 27–29
**Introduction**	4	One or two paragraphs summarizing why this case is unique (**may include** references)	Lines 35–56
**Patient Information**	5a	De-identified patient specific information	Lines 59–62
	5b	Primary concerns and symptoms of the patient	Lines 63–70
	5c	Medical, family, and psycho-social history including relevant genetic information	Lines 59–62
	5d	Relevant past interventions with outcomes	Lines 63–77
**Clinical Findings**	6	Describe significant physical examination (PE) and important clinical findings	Lines 63–74
**Timeline**	7	Historical and current information from this episode of care organized as a timeline	Lines 59–101
**Diagnostic Assessment**	8a	Diagnostic testing (such as PE, laboratory testing, imaging, surveys).	Lines 59–77
	8b	Diagnostic challenges (such as access to testing, financial, or cultural)	Lines 72–80
	8c	Diagnosis (including other diagnoses considered)	Lines 59–77
	8d	Prognosis (such as staging in oncology) where applicable	Lines 59
**Therapeutic Intervention**	9a	Types of therapeutic intervention (such as pharmacologic, surgical, preventive, self-care)	Lines 78-96
	9b	Administration of therapeutic intervention (such as dosage, strength, duration)	Lines 78–96
	9c	Changes in therapeutic intervention (with rationale)	Lines 91–96
**Follow-up and Outcomes**	10a	Clinician and patient-assessed outcomes (if available)	Lines 97–100
	10b	Important follow-up diagnostic and other test results	Lines 97–100
	10c	Intervention adherence and tolerability (How was this assessed?)	Lines 93–100
	10d	Adverse and unanticipated events	Lines 97–100
**Discussion**	11a	A scientific discussion of the strengths AND limitations associated with this case report	Lines 103–133
	11b	Discussion of the relevant medical literature **with references**	Lines 103–122
	11c	The scientific rationale for any conclusions (including assessment of possible causes)	Lines 123–133
	11d	The primary "take-away" lessons of this case report (without references) in a one paragraph conclusion	Lines 135–139
**Patient Perspective**	12	The patient should share their perspective in one to two paragraphs on the treatment(s) they received	N/A
**Informed Consent**	13	Did the patient give informed consent? Please provide if requested	**Yes** ☒ **No** ☐
